# Longitudinal impact of stigma resistance on mental health among individuals with mental disorders

**DOI:** 10.1007/s11136-025-03967-2

**Published:** 2025-04-21

**Authors:** Kevin Ka Shing Chan, Jack Ka Chun Tsui

**Affiliations:** 1 Department of Psychology, The Education University of Hong Kong, Tai Po, Hong Kong; 2 Centre for Psychosocial Health, The Education University of Hong Kong, Tai Po, Hong Kong

**Keywords:** Stigma resistance, Identity affirmation, Valued living, Psychological distress, Personal recovery, Life satisfaction, Mental illness

## Abstract

**Purpose:**

This study evaluated a conceptual model regarding the longitudinal impact of stigma resistance on the mental health of individuals with mental disorders. Specifically, it examined whether stigma resistance is longitudinally associated with reduced psychological distress, improved personal recovery, and enhanced life satisfaction, and tested whether these associations are mediated by increased identity affirmation and heightened valued living.

**Methods:**

A total of 235 individuals with mental disorders completed questionnaire measures of stigma resistance, identity affirmation, valued living, psychological distress, personal recovery, and life satisfaction at baseline (Month 0; M0) and 12 months later (Month 12; M12). The relations among these variables were examined using path analyses and bootstrap analyses.

**Results:**

Path analyses revealed that stigma resistance at M0 was associated with increased identity affirmation and heightened valued living at M12, which, in turn, were associated with reduced psychological distress, improved personal recovery, and enhanced life satisfaction at M12. Bootstrap analyses further demonstrated that stigma resistance at M0 had indirect effects on psychological distress, personal recovery, and life satisfaction at M12 through identity affirmation and valued living at M12.

**Conclusions:**

Theoretically, our findings elucidate how stigma resistance can help individuals with mental disorders improve their mental health by fostering a positive identity and aligning their lives with personal values. Practically, these findings underscore the importance of developing interventions aimed at promoting stigma resistance in these individuals, enabling them to lead more fulfilling and flourishing lives.

**Supplementary Information:**

The online version contains supplementary material available at 10.1007/s11136-025-03967-2.

## Introduction

Public stigma refers to the general public’s tendency to negatively label, stereotype, isolate, and discriminate against individuals with socially discredited attributes [1, 2]. Research shows that public stigma toward individuals with mental disorders is widespread across various geographical and cultural settings [3, 4]. Due to their psychiatric symptoms, these individuals are often misperceived as unpredictable, violent, and incapable [5, 6]. As a result, they may experience a decline in social status and face unequal treatment in key areas of community life, including education, employment, housing, healthcare, and social services [7, 8]. Additionally, they may suffer from verbal abuse, physical violence, and social exclusion in interpersonal interactions [9, 10]. These manifestations of public stigma can create a highly stressful living environment, hindering the process of mental health recovery [11, 12].

When individuals with mental disorders continuously experience public stigma, they may gradually internalize the public’s stigmatizing attitudes and develop self-stigma [13, 14]. Self-stigma refers to the process by which stigmatized individuals adopt, internalize, and integrate the public’s negative views into their own belief systems [15, 16]. Individuals experiencing self-stigma often develop negative thoughts and feelings about themselves and their stigmatized identities [17, 18]. Specifically, they may perceive themselves as inferior and of lesser value than others, leading to reduced self-esteem and self-efficacy [19, 20]. Moreover, they may experience heightened feelings of shame and helplessness due to their psychiatric diagnosis and illness identity [21, 22].

The on-going manifestations of stigma at the societal, community, interpersonal, and intrapersonal levels can significantly hinder individuals with mental disorders from engaging in valued living [23, 24]. Valued living refers to participating in daily activities that align with personal values, fostering a sense of meaning and purpose in life [25, 26]. When individuals with mental disorders constantly face stigma at various socio-ecological levels, they may feel powerless and become socially anxious [27, 28]. This can lead them to actively engage in social withdrawal, ultimately hindering their ability to pursue life opportunities and fully participate in community life [29, 30]. Additionally, when they develop self-stigmatizing beliefs and attitudes, they may consider themselves as incapable or unworthy of achieving their personal goals, which diminishes their motivation to engage in goal-directed behaviors [31, 32]. As a result, they may experience a reduced sense of fulfillment in life [23, 24].

Importantly, the adverse psychological effects of stigma can impair mental health and hinder recovery among individuals with mental disorders [13, 33]. When these individuals struggle to cope with stigmatizing experiences and to live in alignment with their personal values, they may feel disempowered and experience a diminished sense of autonomy and self-determination [34, 35]. Consequently, they may exhibit lower levels of positive emotions and happiness, alongside heightened levels of psychological distress, negative mood, and affective symptoms [36, 37]. Additionally, they may have poorer personal recovery, finding it more difficult to live a hopeful, meaningful, and fulfilling life amidst their mental illness [19, 38]. Furthermore, they may experience reduced life enjoyment and satisfaction [24, 39]. The cumulative effects of these challenges likely include a decline in mental health-related quality of life, characterized by increased negativity toward everyday life (reflecting lower well-being) and diminished capacity to carry out daily activities (indicating poorer functioning) [17, 40].

Given the detrimental impact of stigma on mental health and quality of life, it is crucial to support individuals with mental disorders in developing stigma resistance [41, 42]. Stigma resistance refers to the ability to remain unaffected by public stigma and to actively challenge or deflect encounters with it [43, 44]. Specifically, individuals with stigma resistance are likely to critically reflect on how public stigma devalues, discredits, and marginalizes them [45, 46]. With greater awareness and better understanding of the illegitimacy of public stigma, they are more likely to reject and invalidate stigmatizing views regarding their mental illness [47, 48]. As a result, they are less prone to internalizing public stigma as self-stigma and less susceptible to developing negative self-perceptions or experiencing feelings of shame and inferiority [49, 50].

Research indicates that stigma resistance is more than just the absence of self-stigma [51, 52]. Specifically, it can empower individuals with mental disorders and foster their identity affirmation [41, 48]. Notably, individuals with stigma resistance may take more initiatives to counter negative cultural stereotypes, combat oppressive social structures, and address unequal power dynamics [43, 44]. By engaging in individual and collective actions to confront their disadvantaged situations and advocate for equal rights for their social group, they may recognize their personal capacity to drive social change [43, 44]. This process can enhance their sense of agency and mastery, leading to stronger identity affirmation, characterized by positive attitudes toward their identity as patients [49, 50]. As a result, they may experience greater self-acceptance, self-respect, and self-worth [49, 50].

Stigma resistance can enable individuals with mental disorders to enhance their valued living as well [20, 24]. Specifically, those who exhibit stigma resistance can avoid becoming engulfed in stigma-related memories or overly identifying with self-stigmatizing thoughts and emotions, allowing them to step back and distance themselves from these unpleasant experiences [41, 53]. Instead of getting caught up in negativity through constant rumination and worry, they can let go of their negative experiences and develop a sense of self that is distinct from those experiences [41, 53]. By remaining unaffected by stigma, they can continue to pursue lives and activities that are meaningful and important to them [20, 24]. They can also stay grounded in their long-term aspirations, progressing in life guided by their personal values [20, 24].

By fostering identity affirmation and valued living, stigma resistance can help individuals with mental disorders promote their mental health and quality of life [51, 52]. Individuals with stigma resistance can cope more adaptively with social disapproval and rejection, which enables them to maintain a positive sense of self [41, 42]. They are also better equipped to engage in meaningful roles and pursue activities that hold personal significance [49, 50]. As they become more effective in exerting a positive influence over their own lives, they can experience a greater sense of fulfillment in life [49, 50]. Consequently, they can achieve higher levels of personal recovery and life satisfaction, and experience lower levels of psychological distress, leading to better mental health and improved mental health-related quality of life [41, 48].

### Objectives of the present study

While previous research has primarily focused on the adverse effects of public stigma and self-stigma, limited studies have explored the benefits of cultivating stigma resistance [51, 52]. Indeed, stigma resistance is a distinct construct from public stigma and self-stigma, holding important value on its own due to its significant associations with mental health that extend beyond these other stigma constructs [43, 44]. To date, there has been scant research on the longitudinal impact of stigma resistance on the mental health of individuals with mental disorders, and the mechanisms underlying this impact remain poorly understood [41, 42]. In the present study, we aimed to contribute to the literature by examining whether stigma resistance (*predictor*) prospectively influences identity affirmation and valued living (*mediators*) and, in turn, psychological distress, personal recovery, and life satisfaction (*outcomes*) among individuals with mental disorders. To unravel the temporal dynamics of these variables, we employed a 1-year, two-wave longitudinal research design to explore whether the predictor at Month 0 (M0) is associated with the mediators and outcomes at Month 12 (M12), while controlling for demographic characteristics such as gender, age, and education level at M0, as well as autoregressive effects based on the baseline levels of the mediators and outcomes at M0. This analysis allows us to examine whether the baseline predictor can effectively predict the development of the mediators and outcomes over time. We hypothesized that stigma resistance at M0 would be associated with increased identity affirmation and heightened valued living, leading to reduced psychological distress, improved personal recovery, and enhanced life satisfaction at M12. We also hypothesized that the associations between stigma resistance and the mental health outcomes would be mediated by identity affirmation and valued living.

## Methods

### Participants and procedures

Participants were individuals with mental disorders recruited from four non-governmental organizations in Hong Kong that provide mental health services. Inclusion criteria were having a psychiatric diagnosis according to DSM-5 criteria confirmed by a psychiatrist and being able to read and write in Chinese. Exclusion criteria were having a DSM-5 diagnosis of neurocognitive disorder or intellectual disability and experiencing clinical instability (such as recent hospitalization within the past month). Eligible participants provided written informed consent and completed questionnaires at baseline (M0) and 12 months later (M12). As compensation for their participation, participants received HK$200 (approximately US$26) cash coupons for each assessment. The study received ethical approval from the authors’ institution and was conducted from November 2020 to January 2022.

### Measures

The scales, originally developed in English, were presented to participants in Chinese. These scales were independently translated by two bilingual translators with subject-matter expertise. The translations were reconciled into a single, harmonized version through discussion. This version was back-translated into English by an independent translator to verify fidelity to the original scales. Discrepancies between the original and translated versions were resolved through iterative revisions. Finally, the translated scales were reviewed by the entire research team to ensure linguistic accuracy, cultural appropriateness, and conceptual equivalence.

#### Stigma resistance

The 5-item stigma resistance subscale of the Internalized Stigma of Mental Illness Scale [54] was used to measure participants’ level of stigma resistance at M0. This subscale included items such as “I can have a good, fulfilling life, despite my mental illness”. Participants responded to each item on a 4-point scale, where 1 denoted “strongly disagree” and 4 denoted “strongly agree”. The ratings were averaged, with higher average scores representing greater stigma resistance. Previous research has established the measure’s validity and reliability [41]. In the present study, its Cronbach’s alpha was 0.86.

#### Identity affirmation

The 3-item identity affirmation scale [55] was adapted to measure participants’ level of identity affirmation at both M0 and M12. This scale included items such as “I’m proud to be part of the community of individuals with mental disorders”. Participants responded to each item on a 7-point scale, where 1 denoted “strongly disagree” and 7 denoted “strongly agree”. The ratings were averaged, with higher average scores representing greater identity affirmation. Previous research has established the measure’s validity and reliability [56]. In the present study, its Cronbach’s alphas were 0.86 and 0.82 at M0 and M12, respectively.

#### Valued living

The 5-item valued living subscale of the Engaged Living Scale [25] was used to measure participants’ level of valued living at both M0 and M12. This subscale included items such as “I make choices based on my values, even if it is stressful”. Participants responded to each item on a 5-point scale, where 1 denoted “completely disagree” and 5 denoted “completely agree”. The ratings were averaged, with higher average scores representing greater valued living. Previous research has established the measure’s validity and reliability [24]. In the present study, its Cronbach’s alphas were 0.76 and 0.81 at M0 and M12, respectively.

#### Psychological distress

The 6-item Symptom-Checklist [57] was used to measure participants’ level of psychological distress at both M0 and M12. This scale included items such as “How much were you bothered by feeling blue?”. Participants responded to each item on a 5-point scale, where 1 denoted “not at all” and 5 denoted “extremely”. The ratings were averaged, with higher average scores representing greater psychological distress. Previous research has established the measure’s validity and reliability [58]. In the present study, its Cronbach’s alphas were 0.91 and 0.91 at M0 and M12, respectively.

#### Personal recovery

The 12-item Recovery Assessment Scale [59] was used to measure participants’ level of personal recovery at both M0 and M12. This scale included items such as “I’m hopeful about my future”. Participants responded to each item on a 5-point scale, where 1 denoted “strongly disagree” and 5 denoted “strongly agree”. The ratings were averaged, with higher average scores representing greater personal recovery. Previous research has established the measure’s validity and reliability [60]. In the present study, its Cronbach’s alphas were 0.92 and 0.92 at M0 and M12, respectively.

#### Life satisfaction

The 5-item Satisfaction with Life Scale [61] was used to measure participants’ level of life satisfaction at both M0 and M12. This scale included items such as “I am satisfied with my life”. Participants responded to each item on a 7-point scale, where 1 denoted “strongly disagree” and 7 denoted “strongly agree”. The ratings were averaged, with higher average scores representing higher life satisfaction. Previous research has established the measure’s validity and reliability [60]. In the present study, its Cronbach’s alphas were 0.92 and 0.94 at M0 and M12, respectively.

### Data analyses

Descriptive statistics were calculated for all variables included in the study. To examine the relations among the variables, Pearson’s correlation analyses were performed. To test the hypothesized conceptual model, path analyses were conducted to assess the impact of stigma resistance (*predictor*) at M0 on identity affirmation and valued living (*mediators*) and on psychological distress, personal recovery, and life satisfaction (*outcomes*) at M12, while controlling for demographic factors and autoregressive effects. Missing data were handled using the full information maximum likelihood estimation method to provide relatively unbiased parameter estimates. Model fit was assessed using the Comparative Fit Index (CFI) and the standardized root mean square residual (SRMR). CFI values greater than 0.95 and SRMR values less than 0.08 were indicative of good fit. Lastly, indirect effects within the model were estimated using bias-corrected bootstrap analyses with 1,000 bootstrapped samples. Mediation effects were considered significant if the 95% confidence interval excluded zero. All statistical analyses were conducted using SPSS Version 28.0 and Mplus Version 7.4.

## Results

### Sample characteristics

Table 1 summarizes the sample characteristics. At M0, there were 235 participants, consisting of 54 males and 181 females, with an average age of 47.78 years (*SD* = 12.46 years). The majority of participants had received at least secondary education (94.4%) and were not married (67.7%). Most of them were not in employment (65.1%). The primary psychiatric diagnoses included depressive (43.0%), psychotic (26.0%), bipolar (16.6%), anxiety (13.2%), and obsessive–compulsive (1.3%) disorders. On average, participants had been living with their mental disorders for 14.96 years (*SD* = 10.25 years). The study achieved a good retention rate at M12, with 70.6% (*n* = 166) of participants completing the follow-up assessments. No significant differences in any variables measured at M0 were found between participants who remained in the study and those who dropped out (*p*-values > 0.05).

**Table 1:** Sample characteristics (*n* = 235 at M0; *n* = 166 at M12)

	Sample at M0 (*n* = 235)	Sample at M12 (*n* = 166)
Gender, %		
Male	23.0	21.7
Female	77.0	78.3
Age, years, *M* (SD)	47.78 (12.46)	48.33 (12.24)
Highest education level, %		
Less than primary school	0.4	0.6
Primary school	5.1	6.6
Secondary school	52.3	49.4
College or university	39.1	38.6
Graduate school	3.0	4.8
Marital status, %		
Married	32.3	31.9
Single, separated, divorced, or widowed	67.7	68.1
Employment status, %		
In employment	34.9	40.4
Not in employment	65.1	59.6
Primary diagnosis, %		
Depressive disorder	43.0	47.0
Psychotic disorder	26.0	24.7
Bipolar disorder	16.6	13.3
Anxiety disorder	13.2	13.9
Obsessive compulsive disorder	1.3	1.2
Duration of illness, years, *M* (SD)	14.96 (10.25)	14.98 (10.58)

### Descriptive and correlation analyses

Table 2 summarizes the results of the descriptive and correlation analyses. Notably, there were no significant differences in the variables of interest across different diagnostic groups (*p*-values > 0.05). In the entire sample, all of these variables were significantly correlated with one another (*p*-values < 0.003). Specifically, stigma resistance at M0 was positively correlated with identity affirmation and valued living at both M0 and M12 (*p*-values < 0.003). These variables were negatively correlated with psychological distress at M0 and M12 (*p*-values < 0.001) and positively correlated with personal recovery and life satisfaction at M0 and M12 (*p*-values < 0.001). The effect sizes of these correlations varied from small to large, based on Cohen’s [62] guidelines for interpreting effect size.

**Table 2:** Descriptive statistics of and correlations among variables (*n* = 235 at M0; *n* = 166 at M12)

	*M*	SD	Range	2	3	4	5	6	7	8	9	10	11
1. Stigma resistance (M0)	3.03	0.53	1.00–4.00	0.55^*^**	0.44***	0.63***	0.51***	− 0.56***	− 0.39***	0.72***	0.55***	0.60***	0.42***
2. Identity affirmation (M0)	5.07	1.22	1.00–7.00		0.59***	0.50***	0.24**	− 0.37***	− 0.26***	0.53***	0.35***	0.49***	0.33***
3. Identity affirmation (M12)	5.05	1.15	1.50–7.00			0.35***	0.36***	− 0.32***	− 0.34***	0.39***	0.53***	0.44***	0.43***
4. Valued living (M0)	3.56	0.63	1.00–5.00				0.62***	− 0.54***	− 0.48***	0.70***	0.55***	0.72***	0.54***
5. Valued living (M12)	3.64	0.65	1.60–5.00					− 0.41***	− 0.57***	0.53***	0.72***	0.57***	0.69***
6. Psychological distress (M0)	2.40	0.91	1.00–5.00						0.64***	− 0.67***	− 0.53***	− 0.67***	− 0.52***
7. Psychological distress (M12)	2.42	0.89	1.00–5.00							− 0.49***	− 0.64***	− 0.60***	− 0.65***
8. Personal recovery (M0)	3.70	0.65	1.00–5.00								0.65***	0.67***	0.55***
9. Personal recovery (M12)	3.74	0.63	2.17–5.00									0.65***	0.73***
10. Life satisfaction (M0)	4.28	1.50	1.00–7.00										0.75***
11. Life satisfaction (M12)	4.47	1.55	1.00–7.00										

**p < 0.01

***p < 0.001

### Path analyses

**Fig. 1:**
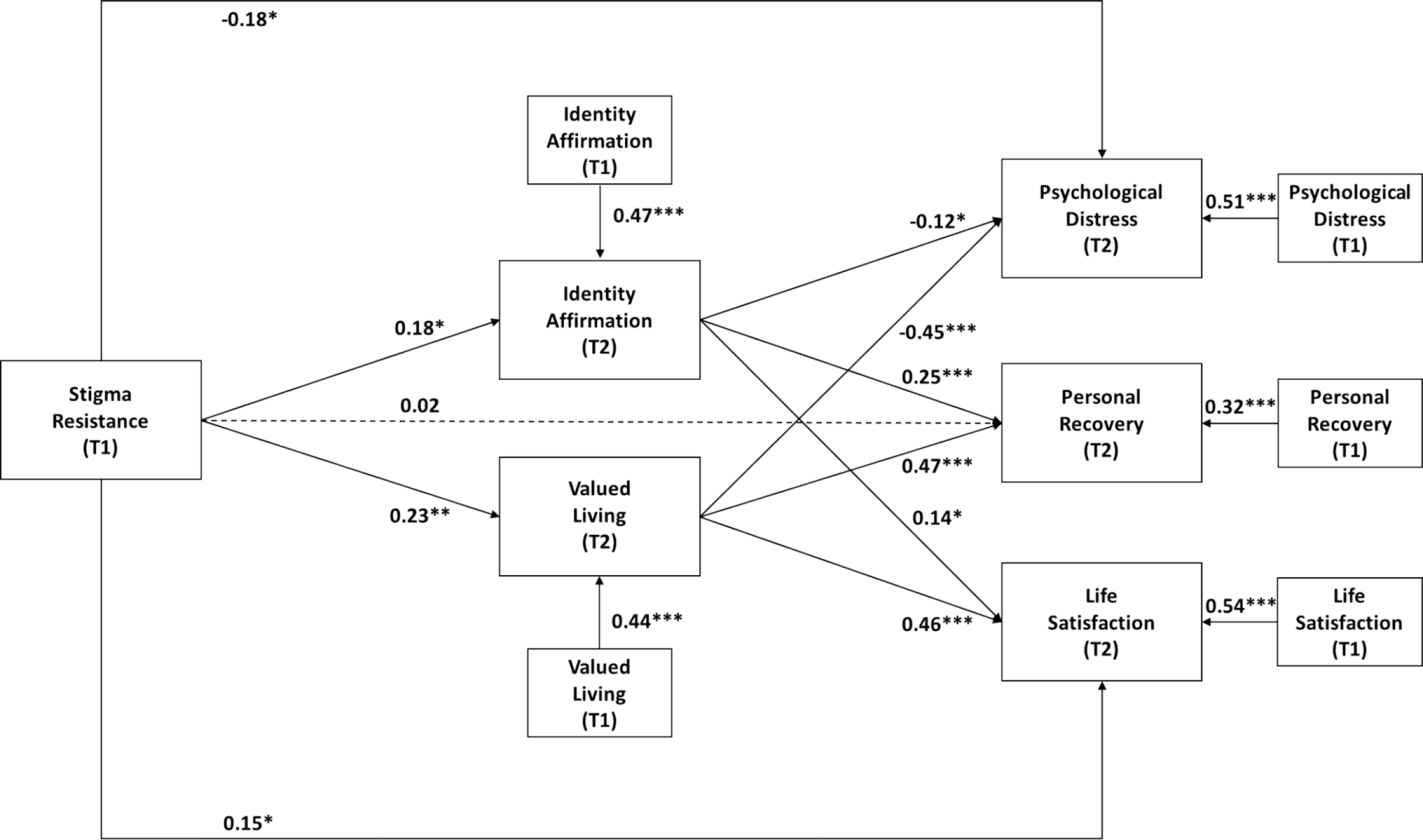
Stigma resistance model for individuals with mental disorders. Demographic factors were included as control variables. Standardized beta coefficients are shown. Solid lines indicate significant paths, whereas dashed lines indicate non-significant paths. *p < 0.05; **p < 0.01; ***p < 0.001

Table 3 summarizes the results of the path analysis. After controlling for demographic factors and autoregressive effects, stigma resistance at M0 had significant direct effects on identity affirmation (*p* = 0.01) and valued living (*p* = 0.002) at M12. Additionally, both identity affirmation and valued living at M12 had significant direct effects on psychological distress (*p*-values < 0.05), personal recovery (*p*-values < 0.001), and life satisfaction (*p*-values < 0.02) at M12. Importantly, even when accounting for the effects of identity affirmation and valued living at M12, stigma resistance at M0 continued to have significant direct effects on psychological distress (*p* = 0.02) and life satisfaction (*p* = 0.01), but not on personal recovery (*p* > 0.05), at M12. The path model shown in Fig. 1 demonstrated a good fit, with a CFI of 0.96 and a SRMR of 0.04. It explained 39.6%, 44.6%, 53.4%, 69.7%, and 67.2% of the variances in identity affirmation, valued living, psychological distress, personal recovery, and life satisfaction at M12, respectively.

**Table 3:** Standardized parameter estimates for the path model (*n* = 235 at M0; *n* = 166 at M12)

			Standardized *β*
*Direct effects*			
Stigma resistance (M0)	→	Identity affirmation (M12)	0.18*
Stigma resistance (M0)	→	Valued living (M12)	0.23**
Stigma resistance (M0)	→	Psychological distress (M12)	− 0.18*
Stigma resistance (M0)	→	Personal recovery (M12)	0.02
Stigma resistance (M0)	→	Life satisfaction (M12)	0.15*
Identity affirmation (M12)	→	Psychological distress (M12)	− 0.12*
Identity affirmation (M12)	→	Personal recovery (M12)	0.25***
Identity affirmation (M12)	→	Life satisfaction (M12)	0.14*
Valued living (M12)	→	Psychological distress (M12)	− 0.45***
Valued living (M12)	→	Personal recovery (M12)	0.47***
Valued living (M12)	→	Life satisfaction (M12)	0.46***
*Autoregressive controls*			
Identity affirmation (M0)	→	Identity affirmation (M12)	0.47***
Valued living (M0)	→	Valued living (M12)	0.44***
Psychological distress (M0)	→	Psychological distress (M12)	0.51***
Personal recovery (M0)	→	Personal recovery (M12)	0.32***
Life satisfaction (M0)	→	Life satisfaction (M12)	0.54***
*Demographic controls*			
Gender (M0)	→	Identity affirmation (M12)	− 0.05
Gender (M0)	→	Valued living (M12)	− 0.13*
Gender (M0)	→	Psychological distress (M12)	− 0.06
Gender (M0)	→	Personal recovery (M12)	0.07
Gender (M0)	→	Life satisfaction (M12)	0.07
Age (M0)	→	Identity affirmation (M12)	0.05
Age (M0)	→	Valued living (M12)	− 0.10
Age (M0)	→	Psychological distress (M12)	− 0.05
Age (M0)	→	Personal recovery (M12)	− 0.12*
Age (M0)	→	Life satisfaction (M12)	− 0.02
Education level (M0)	→	Identity affirmation (M12)	− 0.13*
Education level (M0)	→	Valued living (M12)	− 0.11
Education level (M0)	→	Psychological distress (M12)	− 0.02
Education level (M0)	→	Personal recovery (M12)	− 0.11*
Education level (M0)	→	Life satisfaction (M12)	0.06

*p < 0.05

**p < 0.01

***p < 0.001

### Bootstrap analyses

Table 4 summarizes the results of the bootstrap analysis. Stigma resistance at M0 had significant indirect effects on psychological distress (*p* = 0.003), personal recovery (*p* = 0.001), and life satisfaction (*p* = 0.003) at M12 through the mediating factors of identity affirmation and valued living at M12. Specifically, the indirect effects of stigma resistance at M0 on psychological distress and life satisfaction at M12 were partially mediated by identity affirmation and valued living at M12, while the indirect effects of stigma resistance at M0 on personal recovery at M12 were fully mediated by identity affirmation and valued living at M12.

**Table 4:** Bootstrap analyses for the path model (*n* = 235 at M0; *n* = 166 at M12)

	Standardized indirect effect [95% CI]
*Indirect effects*	
Stigma resistance (M0) → Identity affirmation (M12) and valued living (M12) → Psychological distress (M12)	− 0.12** [− 0.20, − 0.04]
Stigma resistance (M0) → Identity affirmation (M12) and valued living (M12) → Personal recovery (M12)	0.15** [0.06, 0.24]
Stigma resistance (M0) → Identity affirmation (M12) and valued living (M12) → Life satisfaction (M12)	0.13** [0.04, 0.21]

**p < 0.01

## Discussion

In line with our prior hypotheses, our results indicated that stigma resistance was negatively associated with psychological distress and positively associated with personal recovery and life satisfaction, and these associations were mediated by higher levels of identity affirmation and valued living. These results suggest that individuals with greater stigma resistance are better able to affirm their illness identities and lead lives aligned with their personal values. By actively challenging and resisting negative societal judgments, these individuals free themselves from feelings of inferiority and disempowerment, allowing them to cultivate a positive self-perception and pursue a life of personal significance. Ultimately, they achieve higher levels of personal recovery and life satisfaction, along with reduced levels of psychological distress, contributing to better mental health and improved quality of life.

### Longitudinal impact of stigma resistance on mental health outcomes

Previous research has established that when individuals with mental disorders endorse and internalize public stigma as self-stigma, it can lead to negative self-perceptions and heightened psychological distress, thereby hindering their progress toward symptomatic remission and clinical recovery [13, 14]. Expanding on this body of research work, our study revealed a significant negative relation between stigma resistance and psychological distress. This suggests that individuals who actively resist and counteract public stigma may experience lower levels of psychological distress. By actively challenging societal disapproval and devaluation, these individuals may effectively reduce stigma internalization and its associated mental health problems. These findings align with past studies that have indicated the potential of stigma resistance in promoting better psychological well-being, including reduced emotional distress, fewer affective symptoms, and improved clinical recovery [51, 52].

Past research has indicated that stigma resistance plays a pivotal role in empowering individuals with mental disorders to establish a contributing, hopeful, and satisfying life despite the presence of a stigmatized illness identity [41, 49, 50]. Building on these findings, our study revealed positive associations between stigma resistance and both personal recovery and life satisfaction. Specifically, our findings suggest that individuals who actively confront and overcome stigma are better able to enhance their positive perceptions of recovery and promote their subjective quality of life. These positive psychological outcomes of stigma resistance align with prior research demonstrating the associations between stigma resistance and more positive attitudes toward recovery, as well as more favorable evaluations of their own lives [41, 49, 50].

### Mediating roles of identity affirmation and valued living

Stigma resistance was linked to reduced psychological distress and increased personal recovery and life satisfaction through enhanced identity affirmation in individuals with mental disorders. This mediation occurs likely because individuals with stigma resistance are more proactive in countering negative cultural stereotypes, challenging oppressive structures, and addressing power imbalances [43, 44]. By taking actions to advocate for their rights, they recognize their ability to effect social change [43, 44]. This process can foster a sense of agency and mastery, boosting self-appreciation and self-confidence, which further strengthens identify affirmation [49, 50]. In this way, stigma resistance can promote better mental health and improve quality of life for individuals with mental disorders [49, 50].

The longitudinal associations of stigma resistance with psychological distress, personal recovery, and life satisfaction were mediated by valued living as well. This occurs probably because individuals with stigma resistance can demonstrate greater resilience against stigma, which allows them to feel less constrained by the stigma imposed on them [41, 53]. They can also be better equipped to engage in valued life activities, persist in pursuing personally meaningful goals, and align their daily activities with their authentic selves, despite potential discrimination [41, 53]. This process can empower individuals with mental disorders to reclaim their sense of control and autonomy in their lives, enabling them to navigate their own paths to rehabilitation and recovery [41, 53]. Ultimately, this can lead to improved personal recovery, higher life satisfaction, and reduced psychological distress, enhancing mental health-related quality of life [20, 24].

### Stigma resistance model for individuals with mental disorders

The present study represents one of the initial efforts to validate a conceptual model elucidating the longitudinal impact of stigma resistance on mental health in individuals with mental disorders. This model advances the literature by revealing the mediating mechanisms between stigma resistance and mental health. It is noteworthy, however, that the model accounted for only a moderate proportion of the variance in the outcome variables. This result is not entirely unexpected, as the protective benefits of stigma resistance may interact with the negative influences of perceived public stigma and self-stigma, collectively shaping identity affirmation, valued living, and ultimately mental health outcomes [41, 51]. Given the limited research exploring the interplay and combined effects of various stigma-related constructs (e.g., perceived public stigma, self-stigma, and stigma resistance) on mental health, further studies are needed to develop and evaluate a more comprehensive model that addresses these interactions and their cumulative effects.

### Implications for interventions

Given the psychological benefits of stigma resistance, future mental health services should help individuals with mental disorders develop the ability to remain unaffected by public stigma and actively challenge it. Specifically, mental health service providers should assist these individuals in enhancing their awareness of the illegitimacy of public stigma and empower them to undertake anti-stigma efforts [17, 24]. Future researchers should develop evidence-based interventions that enhance stigma resistance in individuals with mental disorders through randomized controlled trials. By intentionally manipulating stigma resistance and observing its changes and effects on mental health outcomes, these trials can further test our conceptual model based on the interventionist theory of causality [63], establishing causal relations among variables and revealing the mechanisms underlying the psychological effects of stigma resistance.

It is important to note that while stigma resistance may help individuals with mental disorders buffer the adverse psychological effects of public stigma, a key long-term question is how to eradicate the roots of public stigma and foster cultural changes in attitudes and behaviors toward individuals with mental disorders. In order to reduce the public’s negative perceptions of mental illness, mental health organizations should conduct community-based psychoeducation programs that challenge biased beliefs about mental illness and replace them with accurate information [2, 64]. Also, to combat the public’s prejudicial attitudes, these organizations should implement contact-based interventions that promote positive interactions between people with and without mental illness [2, 64]. Furthermore, to decrease the public’s discriminatory actions against individuals with mental disorders, mental health organizations should launch large-scale advocacy campaigns that promote the values of diversity and inclusion in society [2, 64].

### Limitations

This study has several limitations. First, our sample consisted solely of Chinese people with mental illness, which may limit the generalizability of our findings to individuals from other ethnic and cultural backgrounds. To test whether our findings can be extrapolated to a broader population, future studies should include samples with greater ethnic and cultural diversity. Second, all our measures relied on self-reports from participants, which may have been influenced by common method and single-reporter biases. Additionally, our measures included translated and adapted scales that require further standardization for the study population. To enhance assessment validity, future studies should employ multi-method, multi-informant approaches alongside fully standardized measures to evaluate the variables. Third, while this study measured stigma resistance at baseline and assessed subsequent changes in mental health outcomes to establish a temporal sequence, the lack of follow-up measurements for stigma resistance limits our ability to account for its potential changes over time, which may also affect mental health outcomes. To gain a more comprehensive understanding of the relations among these variables, future studies should measure all of them at each time point and use cross-lagged analyses to investigate their changes and interrelations.

## Conclusions

Despite these limitations, the present study has made significant contributions to both theoretical understanding and practical applications. Theoretically, our findings elucidate how stigma resistance can help individuals with mental disorders reduce psychological distress, enhance personal recovery, and improve life satisfaction by fostering a positive identity and aligning their lives with personal values. Specifically, the longitudinal nature of these effects highlights that early stigma resistance can provide enduring benefits for individuals with mental disorders over time. Practically, our findings highlight the importance of developing interventions that promote stigma resistance among individuals with mental disorders. Such interventions have the potential to increase identity affirmation, enhance valued living, and ultimately improve the mental health and quality of life of individuals living with mental disorders.

## Supplementary Information

Below is the link to the electronic supplementary material.

Supplementary file1 (DOCX 34 KB)

## Data Availability

The data of this study are available from the corresponding author upon reasonable request.
